# An Evolutionary Model for the Ancient Origins of Polycystic Ovary Syndrome

**DOI:** 10.3390/jcm12196120

**Published:** 2023-09-22

**Authors:** Daniel A. Dumesic, David H. Abbott, Gregorio D. Chazenbalk

**Affiliations:** 1 Department of Obstetrics and Gynecology, David Geffen School of Medicine at UCLA, 10833 Le Conte Ave, Los Angeles, CA 90095, USA; gchazenbalk@gmail.com; 2 Department of Obstetrics and Gynecology, Wisconsin National Primate Research Center, University of Wisconsin, 1223 Capitol Court, Madison, WI 53715, USA; abbott@primate.wisc.edu

**Keywords:** polycystic ovary syndrome, hyperandrogenism, insulin resistance, adipocyte, adipose stem cells, evolution, body fat distribution, metabolic adaptation

## Abstract

Polycystic ovary syndrome (PCOS) is a common endocrinopathy of reproductive-aged women, characterized by hyperandrogenism, oligo-anovulation and insulin resistance and closely linked with preferential abdominal fat accumulation. As an ancestral primate trait, PCOS was likely further selected in humans when scarcity of food in hunter–gatherers of the late Pleistocene additionally programmed for enhanced fat storage to meet the metabolic demands of reproduction in later life. As an evolutionary model for PCOS, healthy normal-weight women with hyperandrogenic PCOS have subcutaneous (SC) abdominal adipose stem cells that favor fat storage through exaggerated lipid accumulation during development to adipocytes in vitro. In turn, fat storage is counterbalanced by reduced insulin sensitivity and preferential accumulation of highly lipolytic intra-abdominal fat in vivo. This metabolic adaptation in PCOS balances energy storage with glucose availability and fatty acid oxidation for optimal energy use during reproduction; its accompanying oligo-anovulation allowed PCOS women from antiquity sufficient time and strength for childrearing of fewer offspring with a greater likelihood of childhood survival. Heritable PCOS characteristics are affected by today’s contemporary environment through epigenetic events that predispose women to lipotoxicity, with excess weight gain and pregnancy complications, calling for an emphasis on preventive healthcare to optimize the long-term, endocrine-metabolic health of PCOS women in today’s obesogenic environment.

## 1. Introduction

As the most common endocrinopathy of reproductive-aged women, polycystic ovary syndrome (PCOS) is characterized by hyperandrogenism, oligo-anovulation and insulin resistance and closely linked with preferential abdominal fat accumulation [1]. Its clinical manifestations of hirsutism, menstrual irregularity, glucose intolerance and dyslipidemia worsen with obesity to increase the risks of developing subfertility, diabetes, metabolic syndrome and/or cardiovascular disease [2]. Almost one half of women with PCOS in the United States have metabolic syndrome (i.e., increased abdominal (android) obesity, hyperglycemia, dyslipidemia and/or hypertension), with a prevalence higher than that of age-matched women without PCOS in this country [1,3] and of PCOS women in other countries where obesity is less common [4,5].

**Figure 1:**
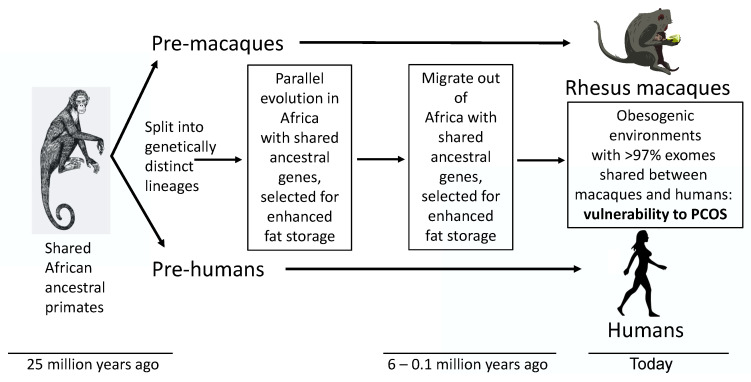
Polycystic ovary syndrome as an ancient metabolic-reproductive adaptation that originally enhanced fat storage for survival of humans during ancient times of food deprivation and also favored fewer offspring with a greater likelihood of childhood survival, but now predisposes women to endocrine-reproductive dysfunction in today’s obesogenic environment (16). Ancestral traits resembling PCOS also exist in female rhesus macaques [8,9,10], which share a common ancestor with humans through parallel evolution.

Through an evolutionary perspective, the high worldwide prevalence of PCOS in today’s environment should have disappeared over millennia, unless a beneficial effect favored both survival and reproduction [6]. Perhaps not surprisingly, therefore, ancestral traits resembling PCOS have been reported throughout antiquity [7] and in a non-human primate (i.e., the female rhesus macaque) [8,9,10] that shares a common ancestor with humans [11]. One explanation is that an ancient female primate trait resembling PCOS may have been favored originally in the cooling, increasingly arid and less forested African environments of the Oligocene before ancestors of humans diverged from those of macaques [12,13], as the isolated continent of Africa contacted Euroasia [14], enabling intercontinental migration [15] (Figure 1).

Such an ancestral trait may have been additionally favored in human hunter–gatherers of the late Pleistocene, or in more ancient human populations, when scarcity of food further selected for programming of enhanced fat storage to meet the metabolic demands of reproduction in later life (i.e., metabolic thrift) [7,16,17,18]. Parallel evolution in macaques, particularly rhesus macaques living in semi-desert and high-altitude environments [13], may have emulated selection in humans for programming of enhanced fat storage (Figure 1). Such evolutionary metabolic adaptations in female primates, including women, would complement the ancient sympathoadrenal response to stress, whereby altered glucocorticoid and catecholamine activities mobilize hepatic glucose and FFAs from visceral fat to act in concert with insulin resistance and ensure sufficient energy during a “fight or flight” response for survival [19,20,21].

Through this evolutionary perspective, the present review examines PCOS as an ancient metabolic adaptation that underwent additional selection pressure for survival of humans during ancient times of food deprivation, but now predisposes to metabolic-endocrine-reproductive dysfunction in today’s obesogenic environment [17,18,22]. A parallel obesogenic environmental change experienced by female rhesus macaques in their natural habitat [13], as well as by macaques removed from their natural habitat decades ago and housed in United States National Primate Research Centers (NPRCs) [23,24], may emulate the current obesogenic environmental challenge confronting humans (Figure 1). Consistent with this notion, approximately 15% of adult female rhesus macaques at the Wisconsin NPRC are naturally hyperandrogenic and exhibit PCOS-like traits [9,10]. Polycystic ovarian syndrome and PCOS-like phenotypes may thus form a continuum of ancient primate traits. Understanding trait-related molecular mechanisms, including genetic, epigenetic, protein and lipid interactions leading to optimal energy utilization, along with the perspective of providing benefits for survival and reproduction in both humans and rhesus macaques, offers novel insight into more effective clinical management for women with PCOS.

## 2. Genetics and Epigenetics of PCOS

The heritability of PCOS has been established by family and twin studies [25,26,27,28]; the prevalence of PCOS in female first-degree relatives of affected women is 20–40% [25,27,29], with monozygotic versus dizygotic twin studies showing the heritability of PCOS as high as 70% [26]. Large genome-wide association studies (GWAS) in cohorts of PCOS women and controls have identified several PCOS-susceptible loci in candidate genes involving gonadotropin secretion/action, androgen biosynthesis/gonadal function, insulin action/metabolism and follicle development [1,30,31,32,33,34,35,36,37,38]. Several PCOS candidate genes are shared among women with differing PCOS phenotypes (i.e., Rotterdam, National Institutes of Health (NIH) criteria, or self-reported) [36]. Some, such as thyroid adenoma associated (*THADA*) and insulin receptor (*INSR*), are associated with metabolic disorders in PCOS and type 2 diabetes mellitus (T2DM) [39], and others with high bioavailable (unbound) circulating T levels [40]. Genetic correlations between PCOS status and components of metabolic syndrome, including childhood obesity, T2DM, and fasting insulin, high-density lipoprotein-cholesterol (HDL-C) as well as triglyceride (TG) levels, further suggest shared genetic and biological origins between these parameters and PCOS [36,38]. That similar PCOS risk genes are expressed in women with PCOS from Chinese and European populations points to the ancient human origins of PCOS [37,38], potentially dating back before the migration of humans out of sub-Saharan Africa 300,000–50,000 years ago or earlier [41,42].

Importantly, women with NIH-defined PCOS have two distinct PCOS subtypes with different genetic heterogeneity: one defined as a “reproductive” group (23% of cases), characterized by higher luteinizing hormone (LH) and sex hormone binding globulin (SHBG) levels with relatively low body mass index (BMI) and insulin levels; the other defined as a “metabolic” group (37% of cases), characterized by higher BMI, glucose and insulin levels, with lower SHBG and LH levels [38,43]. These PCOS subtypes may differ in their developmental origins [43], with their heritability variably interacting with risk-increasing environmental factors to fully explain its prevalence.

Alternatively, rare variants in *DENND1A*, a gene encoding a 1009 amino acid protein with a clathrin-binding domain regulating endosome-mediated endocytosis, receptor cycling and calcium-dependent signaling cascades [44,45], also have been associated with endocrine-metabolic traits in families of daughters with PCOS [46]. A post-transcription form of *DENND1A*, namely DENND1A.v2, is over-expressed in some PCOS women [47,48], with DENND1A.v2 over-expression in human theca cells increasing androgen biosynthesis/release, potentially through PCOS-candidate gene *ZNF217* diminishing the theca cell expression of microRNA mIR-130b-3p, a noncoding microRNA transcriptional repressor [49].

Genetic variants of anti-mullerian hormone (AMH) and its type 2 receptor (AMHR2) also have been identified in about 7% of women with PCOS by NIH criteria, with 37 such variants having reduced in vitro bioactivity and diminished AMH inhibition of CYP17A1 as a risk factor for PCOS [50,51]. Both AMH and AMHR2 gene variants regulate intra-ovarian follicle development and hypothalamic GnRH function, and possibly ovarian androgen production [52], and may underlie elevated circulating AMH levels and ovarian hyperandrogenism in PCOS women [51].

Considered together, the current understanding of the genetics of PCOS suggests multiple contributing risk genes within which different variants can contribute to a PCOS phenotype. Given the heterogeneity of PCOS phenotypic expression, the high prevalence of PCOS, and its complex gene associations that account for some PCOS cases, PCOS may have multiple molecular underpinnings that arise from common or varied developmental origins.

Epigenetic changes coexist with many of these PCOS candidate genes [53,54]. In SC abdominal adipose, over-expression of the LHCG receptor and under-expression of the insulin receptor in non-obese and obese PCOS women, respectively, accompany reciprocal DNA methylation patterns [55], while reciprocal changes of gene expression and DNA methylation also coexist in adipogenic pathways of overweight PCOS women [56]. In PCOS theca cells, moreover, decreased expression of miR-130b-3b (i.e., a noncoding microRNA transcriptional repressor) correlates with increased DENND1A.V2 and CYP17A1 expression as well as with androgen synthesis [49,57], while three PCOS-specific gene variants of AMHR2 occur in regions of higher methylation and acetylation activity [51]. PCOS-susceptible loci alone, however, do not fully explain the majority of PCOS phenotypic expression [58], so that heritability of PCOS likely involves one or more PCOS candidate genes that have interacted with environmental factors throughout antiquity to modify the target tissue phenotype through epigenetic events [5].

## 3. PCOS Phenotypic Expression

Most women with PCOS have systemic insulin resistance from perturbed insulin receptor/post-receptor signaling, altered adipokine secretion and/or abnormal steroid metabolism [2], in combination with preferential abdominal fat accumulation worsened by obesity [1,59,60,61]. Most women with PCOS also have increased adiposity [62,63,64], which interacts with hyperandrogenism to worsen PCOS phenotypic expression [1,2,3,65,66,67] and insulin resistance [2,68,69]. Different PCOS phenotypes according to the Rotterdam criteria also vary in endocrine-metabolic dysfunction [70], with NIH-defined PCOS women (i.e., hyperandrogenism with oligo-anovulation) having the greatest risk of developing menstrual irregularity, anovulatory infertility, T2DM and metabolic syndrome [1]. Furthermore, women with PCOS from a referral population have a more severe phenotype than those from the general population [71,72].

To understand the origins of PCOS, the above variables underlying endocrine-metabolic dysfunction in PCOS need to be eliminated when comparing the clinical characteristics of healthy, normal-weight PCOS women according to the NIH criteria with age/BMI-balanced controls [68,69,71,73,74]. In doing so, healthy normal-weight PCOS women as defined by the NIH criteria show low-normal insulin sensitivity (Si) in frequently sampled intravenous glucose tolerance testing (FSIVGTT) in combination with preferential abdominal fat accumulation (i.e., android fat) as determined by total body dual-energy x-ray absorptiometry (DXA) [59,75,76]. Compared to age- and BMI-matched controls, normal-weight PCOS women as determined by the NIH criteria also exhibit adipose insulin resistance (adipose-IR; defined by the product of fasting circulating free fatty acid (FFA) and insulin levels) [73,76,77].

## 4. Total Abdominal (Android) Fat Mass

Abdominal fat mass comprises two major adipose depots: subcutaneous (SC) and intra-abdominal adipose. In humans, SC abdominal adipose normally stores lipid as protection against insulin resistance, while intra-abdominal adipose has the opposite effect [78]. Total body dual-energy x-ray absorptiometry studies confirm that android fat mass and the percent android fat relative to total body fat are greater in normal-weight PCOS women than age- and BMI-matched controls [59,75]. In all women combined, android fat mass positively correlates with circulating levels of total testosterone (T), free T, androstenedione (A4) and fasting insulin, as does the percent android fat mass relative to total body fat with circulating levels of total T, free T, A4 and fasting insulin [59,76]. Android fat mass in these individuals also negatively correlates with circulating cortisol levels, demonstrating an opposing system interplay of testosterone with cortisol in the control of android fat mass in women with PCOS [76].

Adjusting for fasting insulin levels, android fat mass remains positively correlated with circulating total T levels, as does the percent android fat mass relative to total body fat with circulating levels of total T, free T and A4 [59]. In these normal-weight PCOS women, moreover, androgen receptor blockade by low-dose flutamide simultaneously decreases percent android fat and increases fasting glucose levels, supporting the role of androgen excess in the metabolic adaptation of PCOS through body fat distribution [16,79].

### 4.1. Intra-Abdominal Adipose

Intra-abdominal (visceral) adipose in humans is normally highly lipolytic and resists androgen inhibition of catecholamine-induced lipolysis (lipid breakdown) despite expressing androgen receptors [80]. Intra-abdominal fat mass in normal-weight NIH-defined PCOS women is increased in proportion to circulating androgen concentrations and fasting levels of insulin, TG and non-high-density lipoprotein (non-HDL) cholesterol [59]; it also exhibits exaggerated catecholamine-induced lipolysis in non-obese PCOS women [81,82]. These intra-abdominal fat characteristics favor enhanced FFA availability for hepatic lipid storage and utilization [83]. However, they also promote insulin resistance with obesity when increased FFA availability exceeds the capacity of target tissues to oxidize fat or convert diacylglycerols to triacylglycerols [81,82,84].

### 4.2. Subcutaneous Abdominal Adipose

**Figure 2:**
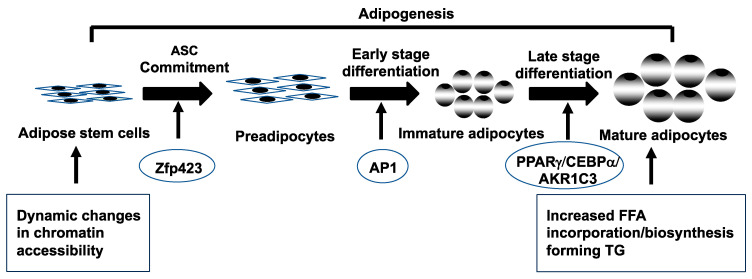
Schematic representation of adipogenesis in subcutaneous abdominal adipose stem cells (ASCs) from normal-weight women with polycystic ovary syndrome. Adipogenesis involves ASC commitment to preadipocytes, followed by an early/late stage preadipocyte differentiation to immature/mature adipocytes [85,86,87]. Dynamic changes in chromatin accessibility of SC abdominal ASCs during adipogenesis activate different transcriptional factors/genes (zinc-finger protein 423 (Zfp423), activator protein-1 (AP-1), peroxisome proliferator-activated receptor *γ* (PPARγ), CCAAT enhancer binding protein *a* (CEBPα) and aldo-ketoreductase type 1C3 (AKR1C3), leading to increased free fatty acid (FFA) incorporation/biosynthesis, thus forming triglycerides (TGs) in newly-formed mature adipocytes. In this manner, SC adipose can increase its fat storage through enlargement of mature adipocytes (i.e., hypertrophy) and development of new adipocytes (i.e., hyperplasia) to buffer fatty acid influx as energy intake exceeds its expenditure [88,89].

Subcutaneous abdominal adipose normally protects against insulin resistance by balancing lipogenesis (lipid formation) with lipolysis (lipid breakdown) in mature adipocytes in combination with adipogenesis (whereby adipose stem cells [ASCs] initially commit to preadipocytes and then differentiate into newly formed adipocytes) (Figure 2) [85,86,87,88,89].

Within SC adipose, androgen normally diminishes insulin-stimulated glucose uptake and impairs catecholamine-stimulated lipolysis through reduced β2-adrenergic receptor and hormone-sensitive lipase (HSL) protein expression [80,81,90]. Women with PCOS have similar SC abdominal adipose characteristics of diminished insulin-mediated glucose uptake, reduced glucose transporter type 4 (GLUT-4) expression [91] and catecholamine lipolytic resistance [92,93]. Importantly, catecholamine lipolytic resistance in normal-weight PCOS women [92,93] can be counterbalanced by impaired insulin suppression of lipolysis in overweight PCOS women [94].

Within SC adipose, an aldo-ketoreductase enzyme, namely aldo-ketoreductase type 1C3 (AKR1C3), generates local T from A4 [95,96]. AKR1C3 gene expression and activity are greater in SC gluteal than omental fat, with SC gluteal fat favoring androgen activation (i.e., AKR1C3), and omental cells favoring androgen inactivation (i.e., aldo-ketoreductase type 1C2 (AKR1C2)) [96]. In PCOS women, increased AKR1C3-mediated androgen activation enhances lipid storage through increased lipogenesis and decreased lipolysis [97,98], promoting fat accretion [75,98,99] despite diminished insulin-stimulated glucose uptake [90].

### 4.3. Subcutaneous Abdominal Stem Cells and Cellular Reprogramming

Subcutaneous abdominal ASCs from normal-weight PCOS women exhibit altered dynamic chromatin accessibility during adipogenesis compared to control ASCs and are characterized by limited chromatin accessibility in undifferentiated ASCs (quiescent stage) followed by exaggerated availability (active stage) in newly-formed adipocytes [100]. These chromatin remodeling patterns of PCOS stem cells accompany enrichment of binding motifs for transcription factors (TFs) of the activator protein-1 (AP-1) subfamily during early cell differentiation, with altered gene expression of adipogenic/angiogenic functions involving androgen–insulin interactions through transforming growth factor (TGF)-ß1 signaling [77].

In these SC abdominal ASCs of normal-weight PCOS women, an exaggerated commitment to preadipocytes via zinc-finger protein 423 (*ZFP423*) overexpression negatively correlates with fasting circulating glucose levels [99] and accompanies a greater proportion of small SC abdominal adipocytes [59,77], presumably to buffer against fatty acid influx [89,101]. Similar small SC abdominal adipocytes occur in other individuals [101,102,103], in whom they protect against insulin resistance through stem cell *ZFP423* upregulation from epigenetic modifications [104].

Following exaggerated commitment to preadipocytes, these same abdominal ASCs from normal-weight PCOS exhibit accelerated lipid accumulation in newly-formed adipocytes in vitro that predicts reduced serum FFA levels and improved systemic insulin sensitivity in vivo [75,99]. These differentiating PCOS stem cells can overexpress the genes, peroxisome proliferator-activated receptor *γ* (*PPARγ*) and CCAAT enhancer binding protein *a* (*CEBPa*), in combination with increased *AKR1C3* gene expression during adipocyte maturation in vitro (Figure 2) [79,98,100].

From a causal perspective, administration of flutamide (an androgen receptor blocker) to healthy normal-weight PCOS women attenuates accelerated lipid accumulation within these newly-formed adipocytes in vitro and increases fasting circulating glucose levels (but within the normal range) [79]. In addition to intrinsic changes in PCOS stem cell characteristics, therefore, local androgen excess in PCOS appears to enhance lipid storage in SC abdominal adipocytes [79,98,99] and favor insulin sensitivity [75,105,106].

## 5. Lipotoxicity

Lipotoxicity refers to the ectopic lipid accumulation in non-adipose tissue, where it induces oxidative/endoplasmic reticulum stress linked with insulin resistance and inflammation [107]. Overweight/obese PCOS women, with greater preferential abdominal fat accumulation, hyperandrogenism and insulin resistance [2], are at particular risk of developing lipotoxicity due to excess FFA uptake into non-adipose cells, in part from increased highly lipolytic intra-abdominal fat with impaired insulin suppression of lipolysis [81,82,94,108,109,110]. In these individuals, excess fatty acid influx in the skeletal muscle and liver promotes diacylglycerol-induced insulin resistance, impairs insulin signaling via increased insulin receptor serine phosphorylation, and disrupts mitochondrial oxidative phosphorylation [84,111]. Enlarged SC abdominal mature adipocytes in overweight compared to normal-weight PCOS women also fosters a pro-inflammatory lipid depot environment [59,94].

Within today’s contemporary lifestyle, NIH-defined PCOS women have a two- to three-fold higher prevalence of metabolic syndrome (33–47%) than age-matched women without PCOS [3,112,113,114], which is reduced by diminished abdominal fat accumulation [114]. Beginning in adolescence, an increased risk for developing metabolic syndrome [115] is evident in hyperandrogenic women [116], who preferentially increase abdominal adiposity with weight gain [61].

Increased abdominal fat in PCOS women also increases the risk of developing nonalcoholic fatty liver disease (NAFLD) [117,118,119], with non-alcoholic hepatic steatosis varying in inflammation and fibrosis [120]. Obesity in PCOS women is an important risk factor for hepatic steatosis [117], as is androgen excess per se, since the probability of hepatic steatosis (37%) and elevated serum aminotransferase levels is greater in hyperandrogenic women with PCOS than age- and weight-matched controls [121,122]. Magnetic resonance spectroscopy further confirms greater liver fat content in women with hyperandrogenic PCOS than non-hyperandrogenic PCOS [123].

## 6. Parallel Evolution of PCOS-like Traits in Naturally Hyperandrogenic Female Rhesus Macaques

Ancestors of macaques migrated out of Africa before humans (Figure 1), about 5–6 million years ago [12,15]. Second only to humans, contemporary rhesus macaques occupy the largest habitat range of any primate, somewhat emulating humans in their diversity of habitats, including obesogenic urban environments [13]. Such close evolutionary history to humans bestows considerable similarities in genomic, developmental, physiological, anatomical, neurological, behavioral and aging characteristics, as well as comparable breadth of natural disease susceptibility [10], including female hyperandrogenism, PCOS [8,9] and obesity [124]. Obesity in rhesus macaques is heritable [125], emulates that in humans [126,127,128] and may associate with human obesity risk genes [125], increased risk of T2DM [127,129], dyslipidemia [12,126,128,130] and cardiometabolic disease [131,132]. In female rhesus macaques, as in women, hyperandrogenism enhances obesity outcomes [128,130,133]. Examining the etiology for female rhesus macaque hyperandrogenism and accompanying PCOS-like traits, including metabolic dysfunction, may thus provide supportive evidence for parallel evolution of these traits to humans and for a shared vulnerability to PCOS (Figure 1). In addition, female rhesus macaques and humans share menstrual cycle traits, including a relatively lengthy follicular or preovulatory phase, exposing selection of a single preovulatory follicle to hyperandrogenic anovulatory consequences of prolonged LH hypersecretion, FSH hyposecretion [134] and hyperinsulinemia [10].

Hyperandrogenic female rhesus monkeys with increased adiposity also emulate the metabolic dysfunction seen in women with PCOS. They exhibit increased abdominal subcutaneous and visceral adiposity [128,133,135], adipose insulin resistance and impaired insulin secretion [136], and an increased incidence of T2DM [137]. Their SC abdominal adipocytes demonstrate an altered ability to store fat relative to BMI [128,130,135,138], with impaired preadipocyte differentiation into adipocytes accompanying a decrease in C/EBPα mRNA. An associated enhancement of SC abdominal ASC commitment to preadipocytes through increased ZFP423 mRNA expression may indicate a compensatory mechanism for impaired preadipocyte differentiation [138]. Those with the highest testosterone values demonstrate increased BMI, central adiposity and insulin resistance [8,128].

Hyperandrogenism in female rhesus monkeys may have developmental origins, emulating PCOS in women. A positive correlation of adult anogenital distance with circulating testosterone levels in naturally hyperandrogenic adult female rhesus monkeys suggests mid-gestational hyperandrogenic origins [9]. Increased anogenital distance has also been reported for girls born to women with PCOS [139], in women with PCOS [140] and in adult female PCOS-like rhesus monkeys previously exposed to early-to-mid, but not late, gestational testosterone excess [141]. Elevated maternal circulating levels of AMH from polycystic ovaries may enhance maternal hyperandrogenism and amplify epigenetic transgenerational transmission of hyperandrogenic and metabolic phenotypes in female offspring through altered placental function [142,143]. Consistent with these findings, gestational hyperandrogenism in rhesus monkeys induces maternal hyperinsulinemia and hyperglycemia and reliably generates 75% of female offspring with heterogenous PCOS-like reproductive and metabolic phenotypes [144], along with gestational hyperinsulinemia inducing ectopic pericardial and perirenal fetal lipid accumulation [145]. Commonly occurring placental structure and function alterations found in women with PCOS [146,147,148,149] and in hyperandrogenic adult female rhesus monkeys [150] can alter nutrient delivery to the fetus [146,151], with subsequent hyperandrogenism, insulin resistance and pancreatic beta cell dysfunction in prepubertal daughters [152,153,154,155], predisposing them to preferential fat storage [138,153]. Given these findings implicating hyperandrogenic developmental origins in the etiology of preferential fat storage, female rhesus monkeys may provide unique insight into the proximate mechanisms amplifying outcomes from the inheritance of PCOS risk genes, calling for gene editing studies of monkey embryos/cells to express female phenotypes generated by specific PCOS risk genes in individuals of known genetic backgrounds [10,156].

## 7. Conclusions

Polycystic ovary syndrome has persisted from antiquity to become the most common endocrine-metabolic disorder of reproductive-aged women. While its ancestral traits once favored abdominal fat deposition and increased energy availability through hyperandrogenism and insulin resistance for reproduction within hostile environments of food deprivation, these same traits now underlie different PCOS phenotypes, with various risks for endocrine-metabolic dysfunction, which are worsened by obesity. Normal-weight women with NIH-defined PCOS who are otherwise healthy have SC abdominal adipose characteristics that favor lipid storage in combination with low-normal insulin sensitivity accompanied by increased highly lipolytic intra-abdominal fat deposition. As an ancestral trait programmed by genetic inheritance and epigenetic amplification during gestation, such an evolutionary metabolic adaptation in normal-weight PCOS women balances enhanced SC adipose storage with increased circulating glucose availability and free fatty acid oxidation as energy substrate for the brain, muscle and other crucial target tissues. This metabolic adaptation in hyperandrogenic PCOS women also favors oligo-ovulation, which allowed women from antiquity sufficient time for childrearing of fewer offspring, who in turn had a greater likelihood of childhood survival [6].

Important strengths of this review paper are the inclusion of normal-weight PCOS women as defined by the NIH criteria, who were otherwise healthy and who were also age- and BMI-matched to controls whenever possible to eliminate the confounding effects of age and obesity on outcomes of interest. It is important to recognize, however, that this review is not intended to be a comprehensive review of the field of PCOS. Rather, it explores the hypothesis, based on the available (epi)genetic and physiological data, that the phenotypic expression of PCOS represents an evolutionary metabolic adaptation that balances preferential abdominal fat accumulation with increased energy availability through hyperandrogenism and insulin resistance to optimize energy use for reproduction during ancient times of food deprivation.

## 8. Future Directions

These heritable PCOS characteristics are now adversely affected by today’s contemporary environment through epigenetic events that predispose women to lipotoxicity, with excess weight gain and pregnancy complications. Understanding the evolutionary origins of PCOS emphasizes the need for a greater focus on preventive healthcare, with early and appropriate lifestyle as well as therapeutic choices to optimize the long-term, endocrine-metabolic health of PCOS women in today’s obesogenic environment.

## Data Availability

Not applicable.

## References

[1] Chang R.J., Dumesic D.A., Strauss J.F., Barbieri R.L., Dokras A., Williams C.J., Williams S.Z. (2024). Polycystic Ovary Syndrome and Hyperandrogenic States. Yen and Jaffe’s Reproductive Endocrinology: Physiology, Pathophysiology and Clinical Management.

[2] Dumesic D.A., Oberfield S.E., Stener-Victorin E., Marshall J.C., Laven J.S., Legro R.S. (2015). Scientific Statement on the Diagnostic Criteria, Epidemiology, Pathophysiology, and Molecular Genetics of Polycystic Ovary Syndrome. Endocr. Rev..

[3] Moran L.J., Misso M.L., Wild R.A., Norman R.J. (2010). Impaired glucose tolerance, type 2 diabetes and metabolic syndrome in polycystic ovary syndrome: A systematic review and meta-analysis. Hum. Repro. Update.

[4] Carmina E., Napoli N., Longo R.A., Rini G.B., Lobo R.A. (2006). Metabolic syndrome in polycystic ovary syndrome (PCOS): Lower prevalence in southern Italy than in the USA and the influence of criteria for the diagnosis of PCOS. Eur. J. Endocrinol..

[5] Dumesic D.A., Hoyos L.R., Chazenbalk G.D., Naik R., Padmanabhan V., Abbott D.H. (2020). Mechanisms of Intergenerational Transmission of Polycystic Ovary Syndrome. Reproduction.

[6] Corbett S., Morin-Papunen L. (2013). Polycystic ovary syndrome and recent human evolution. Mol. Cell. Endocrinol..

[7] Azziz R., Dumesic D.A., Goodarzi M. (2011). Polycystic Ovary Syndrome: An ancient disorder?. Fertil. Steril..

[8] Arifin E., Shively C.A., Register T.C., Cline J.M. (2008). Polycystic ovary syndrome with endometrial hyperplasia in a cynomolgus monkey (*Macaca fascicularis*). Vet. Pathol..

[9] Abbott D.H., Rayome B.H., Dumesic D.A., Lewis K.C., Edwards A.K., Wallen K., Wilson M.E., Appt S.E., Levine J.E. (2017). Clustering of PCOS-like traits in naturally hyperandrogenic female rhesus monkeys. Hum. Reprod..

[10] Abbott D.H., Rogers J., Dumesic D.A., Levine J.E. (2019). Naturally Occurring and Experimentally Induced Rhesus Macaque Models for Polycystic Ovary Syndrome: Translational Gateways to Clinical Application. Med. Sci..

[11] Perelman P., Johnson W.E., Roos C., Seuánez H.N., Horvath J.E., Moreira M.A., Kessing B., Pontius J., Roelke M., Rumpler Y. (2011). A molecular phylogeny of living primates. PLoS Genet..

[12] Raaum R.L., Sterner K.N., Noviello C.M., Stewart C.B., Disotell T.R. (2005). Catarrhine primate divergence dates estimated from complete mitochondrial genomes: Concordance with fossil and nuclear DNA evidence. J. Hum. Evol..

[13] Cooper E.B., Brent L.J.N., Snyder-Mackler N., Singh M., Sengupta A., Khatiwada S., Malaivijitnond S., Qi Hai Z., Higham J.P. (2022). The rhesus macaque as a success story of the Anthropocene. Elife.

[14] Leakey M., Grossman A., Gutiérrez M., Fleagle J.G. (2011). Faunal change in the Turkana Basin during the late Oligocene and Miocene. Evol. Anthropol..

[15] Stewart C.B., Disotell T.R. (1998). Primate evolution—In and out of Africa. Curr. Biol..

[16] Dumesic D.A., Padmanabhan V., Levine J., Chazenbalk G.D., Abbott D.H. (2022). Polycystic Ovary Syndrome as a Plausible Evolutionary Metabolic Adaptation. Repro. Biol. Endocrinol..

[17] Parker J., O’Brien C., Hawrelak J., Gersh F.L. (2022). Polycystic Ovary Syndrome: An Evolutionary Adaptation to Lifestyle and the Environment. Int. J. Environ. Res. Public Health.

[18] Parker J. (2023). Pathophysiological Effects of Contemporary Lifestyle on Evolutionary-Conserved Survival Mechanisms in Polycystic Ovary Syndrome. Life.

[19] Tsatsoulis A., Mantzaris M.D., Bellou S., Andrikoula M. (2013). Insulin resistance: An adaptive mechanism becomes maladaptive in the current environment—An evolutionary perspective. Metabolism.

[20] Björntorp P. (1991). Metabolic implications of body fat distribution. Diabetes Care.

[21] Björntorp P. (1993). Visceral obesity: A “civilization syndrome”. Obes. Res..

[22] López-Otín C., Kroemer G. (2021). Hallmarks of Health. Cell.

[23] Klimentidis Y.C., Beasley T.M., Lin H.Y., Murati G., Glass G.E., Guyton M., Newton W., Jorgensen M., Heymsfield S.B., Kemnitz J. (2011). Canaries in the coal mine: A cross-species analysis of the plurality of obesity epidemics. Proc. Biol. Sci..

[24] Terasawa E., Kurian J.R., Keen K.L., Shiel N.A., Colman R.J., Capuano S.V. (2012). Body weight impact on puberty: Effects of high-calorie diet on puberty onset in female rhesus monkeys. Endocrinology.

[25] Legro R.S., Driscoll D., Strauss J.F., Fox J., Dunaif A. (1998). Evidence for a genetic basis for hyperandrogenemia in polycystic ovary syndrome. Proc. Natl. Acad. Sci. USA.

[26] Vink J.M., Sadrzadeh S., Lambalk C.B., Boomsma D.I. (2006). Heritability of polycystic ovary syndrome in a Dutch twin-family study. J. Clin. Endocrinol. Metab..

[27] Risal S., Pei Y., Lu H., Manti M., Fornes R., Pui H.P., Zhao Z., Massart J., Ohlsson C., Lindgren E. (2019). Prenatal androgen exposure and transgenerational susceptibility to polycystic ovary syndrome. Nat. Med..

[28] Shan D., Han J., Cai Y., Zou L., Xu L., Shen Y. (2022). Reproductive Health in First-degree Relatives of Patients with Polycystic Ovary Syndrome: A Review and Meta-analysis. J. Clin. Endocrinol. Metab..

[29] Kahsar-Miller M.D., Nixon C., Boots L.R., Go R.C., Azziz R. (2001). Prevalence of polycystic ovary syndrome (PCOS) in first-degree relatives of patients with PCOS. Fertil. Steril..

[30] Chen Z.J., Zhao H., He L., Shi Y., Qin Y., Shi Y., Li Z., You L., Zhao J., Liu J. (2011). Genome-wide association study identifies susceptibility loci for polycystic ovary syndrome on chromosome 2p16.3, 2p21 and 9q33.3. Nat. Genet..

[31] Shi Y., Zhao H., Shi Y., Cao Y., Yang D., Li Z., Zhang B., Liang X., Li T., Chen J. (2012). Genome-wide association study identifies eight new risk loci for polycystic ovary syndrome. Nat. Genet..

[32] Goodarzi M.O., Jones M.R., Li X., Chua A.K., Garcia O.A., Chen Y.D., Krauss R.M., Rotter J.I., Ankener W., Legro R.S. (2012). Replication of association of DENND1A and THADA variants with polycystic ovary syndrome in European cohorts. J. Med. Genet..

[33] Mutharasan P., Galdones E., Penalver Bernabe B., Garcia O.A., Jafari N., Shea L.D., Woodruff T.K., Legro R.S., Dunaif A., Urbanek M. (2013). Evidence for chromosome 2p16.3 polycystic ovary syndrome susceptibility locus in affected women of European ancestry. J. Clin. Endocrinol. Metab..

[34] Hayes M.G., Urbanek M., Ehrmann D.A., Armstrong L.L., Lee J.Y., Sisk R., Karaderi T., Barber T.M., McCarthy M.I., Franks S. (2015). Genome-wide association of polycystic ovary syndrome implicates alterations in gonadotropin secretion in European ancestry populations. Nat. Commun..

[35] Day F.R., Hinds D.A., Tung J.Y., Stolk L., Styrkarsdottir U., Saxena R., Bjonnes A., Broer L., Dunger D.B., Halldorsson B.V. (2015). Causal mechanisms and balancing selection inferred from genetic associations with polycystic ovary syndrome. Nat. Commun..

[36] Day F., Karaderi T., Jones M.R., Meun C., He C., Drong A., Kraft P., Lin N., Huang H., Broer L. (2018). Large-scale genome-wide meta-analysis of polycystic ovary syndrome suggests shared genetic architecture for different diagnosis criteria. PLoS Genet..

[37] Dapas M., Dunaif A. (2020). The contribution of rare genetic variants to the pathogenesis of polycystic ovary syndrome. Curr. Opin. Endocr. Metab. Res..

[38] Dapas M., Dunaif A. (2022). Deconstructing a Syndrome: Genomic Insights into PCOS Causal Mechanisms and Classification. Endocr. Rev..

[39] Tian Y., Li J., Su S., Cao Y., Wang Z., Zhao S., Zhao H. (2020). PCOS-GWAS Susceptibility Variants in *THADA*, *INSR*, *TOX3*, and *DENND1A* Are Associated with Metabolic Syndrome or Insulin Resistance in Women with PCOS. Front. Endocrinol..

[40] Ruth K.S., Day F.R., Tyrrell J., Thompson D.J., Wood A.R., Mahajan A., Beaumont R.N., Wittemans L., Martin S., Busch A.S. (2020). Using human genetics to understand the disease impacts of testosterone in men and women. Nat. Med..

[41] Shriner D., Tekola-Ayele F., Adeyemo A., Rotimi C.N. (2016). Ancient Human Migration after Out-of-Africa. Sci. Rep..

[42] Nielsen R., Akey J.M., Jakobsson M., Pritchard J.K., Tishkoff S., Willerslev E. (2017). Tracing the peopling of the world through genomics. Nature.

[43] Dapas M., Lin F.T.J., Nadkarni G.N., Sisk R., Legro R.S., Urbanek M., Hayes M.G., Dunaif A. (2020). Distinct subtypes of polycystic ovary syndrome with novel genetic associations: An unsupervised, phenotypic clustering analysis. PLoS Med..

[44] Strauss J.F., McAllister J.M., Urbanek M. (2012). Persistence pays off for PCOS gene prospectors. J. Clin. Endocrinol. Metab..

[45] Tee M.K., Speek M., Legeza B., Modi B., Teves M.E., McAllister J.M., Strauss J.F., Miller W.L. (2016). Alternative splicing of DENND1A, a PCOS candidate gene, generates variant 2. Mol. Cell. Endocrinol..

[46] Dapas M., Sisk R., Legro R.S., Urbanek M., Dunaif A., Hayes M.G. (2019). Family-based quantitative trait meta-analysis implicates rare noncoding variants in DENND1A in polycystic ovary syndrome. J. Clin. Endocrinol. Metab..

[47] McAllister J.M., Modi B., Miller B.A., Biegler J., Bruggeman R., Legro R.S., Strauss J.F. (2014). Overexpression of a DENND1A isoform produces a polycystic ovary syndrome theca phenotype. Proc. Natl. Acad. Sci. USA.

[48] McAllister J.M., Legro R.S., Modi B.P., Strauss J.F. (2015). Functional genomics of PCOS: From GWAS to molecular mechanisms. Trends Endocrinol. Metab..

[49] Waterbury J.S., Teves M.E., Gaynor A., Han A.X., Mavodza G., Newell J., Strauss J.F., McAllister J.M. (2022). The PCOS GWAS Candidate Gene *ZNF217* Influences Theca Cell Expression of *DENND1A.V2*, *CYP17A1*, and Androgen Production. J. Endocr. Soc..

[50] Gorsic L.K., Kosova G., Werstein B., Sisk R., Legro R.S., Hayes M.G., Teixeira J.M., Dunaif A., Urbanek M. (2017). Pathogenic Anti-Mullerian Hormone Variants in Polycystic Ovary Syndrome. J. Clin. Endocrinol. Metab..

[51] Gorsic L.K., Dapas M., Legro R.S., Hayes M.G., Urbanek M. (2019). Functional Genetic Variation in the Anti-Mullerian Hormone Pathway in Women with Polycystic Ovary Syndrome. J. Clin. Endocrinol. Metab..

[52] Barbotin A.L., Peigné M., Malone S.A., Giacobini P. (2019). Emerging Roles of Anti-Müllerian Hormone in Hypothalamic-Pituitary Function. Neuroendocrinology.

[53] Nilsson E., Benrick A., Kokosar M., Krook A., Lindgren E., Källman T., Martis M.M., Højlund K., Ling C., Stener-Victorin E. (2018). Transcriptional and epigenetic changes influencing skeletal muscle metabolism in women with polycystic ovary syndrome. J. Clin. Endocrinol. Metab..

[54] Vázquez-Martínez E.R., Gómez-Viais Y.I., García-Gómez E., Reyes-Mayoral C., Reyes-Muñoz E., Camacho-Arroyo I., Cerbón M. (2019). DNA methylation in the pathogenesis of polycystic ovary syndrome. Reproduction.

[55] Jones M.R., Brower M.A., Xu N., Cui J., Mengesha E., Chen Y.D., Taylor K.D., Azziz R., Goodarzi M.O. (2015). Systems Genetics Reveals the Functional Context of PCOS Loci and Identifies Genetic and Molecular Mechanisms of Disease Heterogeneity. PLoS Genet..

[56] Kokosar M., Benrick A., Perfilyev A., Fornes R., Nilsson E., Maliqueo M., Behre C.J., Sazonova A., Ohlsson C., Ling C. (2016). Epigenetic and Transcriptional Alterations in Human Adipose Tissue of Polycystic Ovary Syndrome. Sci. Rep..

[57] McAllister J.M., Han A.X., Modi B.P., Teves M.E., Mavodza G.R., Anderson Z.L., Shen T., Christenson L.K., Archer K.J., Strauss J.F. (2019). miRNA Profiling Reveals miRNA-130b-3p Mediates DENND1A Variant 2 Expression and Androgen Biosynthesis. Endocrinology.

[58] Abbott D.H., Dumesic D.A., Levine J.E. (2019). Hyperandrogenic Origins of Polycystic Ovary Syndrome—Implications for Pathophysiology and Therapy. Expert Rev. Endocrinol. Metab..

[59] Dumesic D.A., Akopians A.L., Madrigal V.K., Ramirez E., Margolis D.J., Sarma M.K., Thomas A.M., Grogan T.R., Haykal R., Schooler T.A. (2016). Hyperandrogenism Accompanies Increased Intra-Abdominal Fat Storage in Normal Weight Polycystic Ovary Syndrome Women. J. Clin. Endocrinol. Metab..

[60] Tosi F., Di Sarra D., Kaufman J.M., Bonin C., Moretta R., Bonoro E., Zanolin E., Mogetti P. (2015). Total body fat and central fat mass independently predict insulin resistance but not hyperandrogenemia in women with polycystic ovary syndrome. J. Clin. Endocrinol. Metab..

[61] Holte J., Bergh T., Berne C., Berglund L., Lithell H. (1994). Enhanced early insulin response to glucose in relation to insulin resistance in women with polycystic ovary syndrome and normal glucose tolerance. J. Clin. Endocrinol. Metab..

[62] Rosenzweig J.L., Ferrannini E., Grundy S.M., Haffner S.M., Heine R.J., Horton E.S., Kawamori R. (2008). Primary prevention of cardiovascular disease and type 2 diabetes in patients at metabolic risk: An endocrine society clinical practice guideline. J. Clin. Endocrinol. Metab..

[63] Wyatt H.R. (2013). Update on treatment strategies for obesity. J. Clin. Endocrinol. Metab..

[64] Pasquali R., Pelusi C., Genghini S., Cacciari M., Gambineri A. (2003). Obesity and reproductive disorders in women. Hum. Reprod. Update.

[65] Diamanti-Kandarakis E., Dunaif A. (2012). Insulin resistance and the polycystic ovary syndrome revisited: An update on mechanisms and implications. Endocr. Rev..

[66] Lim S.S., Norman R.J., Davies M.J., Moran L.J. (2013). The effect of obesity on polycystic ovary syndrome: A systematic review and meta-analysis. Obes. Rev..

[67] Yildiz B.O., Knochenhauer E.S., Azziz R. (2008). Impact of obesity on the risk for polycystic ovary syndrome. J. Clin. Endocrinol. Metab..

[68] Kakoly N.S., Khomami M.B., Joham A.E., Corray S.D., Misso M.L., Norman R.J., Harrison C.L., Ranasinha S., Teede H.J., Moran L.J. (2018). Ethnicity, obesity and the prevalence of impaired glucose tolerance and type 2 diabetes in PCOS: A systematic review and meta-regression. Hum. Reprod. Update.

[69] Palaniappan L.P., Carnethon M.R., Fortmann S.P. (2002). Heterogeneity in the relationship between ethnicity, BMI, and fasting insulin. Diabetes Care.

[70] Teede H.J., Tay C.T., Laven J., Dokras A., Moran L.J., Piltonen T.T., Costello M.F., Boivin J., Redman L.M., Boyle J.A. (2023). Recommendations from the 2023 International Evidence-based Guideline for the Assessment and Management of Polycystic Ovary Syndrome. Fertil. Steril..

[71] Ezeh U., Yildiz B.O., Azziz R. (2013). Referral bias in defining the phenotype and prevalence of obesity in polycystic ovary syndrome. J. Clin. Endocrinol. Metab..

[72] Lizneva D., Kirubakaran R., Mykhalchenko K., Suturina L., Chernukha G., Diamond M.P., Azziz R. (2016). Phenotypes and body mass in women with polycystic ovary syndrome identified in referral versus unselected populations: Systematic review and meta-analysis. Fertil. Steril..

[73] Søndergaard E., Espinosa De Ycaza A.E., Morgan-Bathke M., Jensen M.D. (2017). How to measure adipose tissue insulin sensitivity. J. Clin. Endocrinol. Metab..

[74] Hershkop K., Besor O., Santoro N., Pierpont B., Caprio S., Weiss R. (2016). Adipose insulin resistance in obese adolescents across the spectrum of glucose tolerance. J. Clin. Endocrinol. Metab..

[75] Dumesic D.A., Tulberg A., Leung K.L., Fisch S.C., Grogan T.R., Abbott D.H., Naik R., Chazenbalk G.D. (2021). Accelerated subcutaneous abdominal stem cell adipogenesis predicts insulin sensitivity in normal-weight women with polycystic ovary syndrome. Fertil. Steril..

[76] Dumesic D.A., Turcu A.F., Liu H., Grogan T.R., Abbott D.H., Lu G., Dharanipragada D., Chazenbalk G.D. (2023). Interplay of Cortisol, Testosterone, and Abdominal Fat Mass in Normal-weight Women with Polycystic Ovary Syndrome. J. Endocr. Soc..

[77] Dumesic D.A., Phan J.D., Leung K.L., Grogan T.R., Ding X., Li X., Hoyos L.R., Abbott D.H., Chazenbalk G.D. (2019). Adipose Insulin Resistance in Normal-Weight Polycystic Ovary Syndrome Women. J. Clin. Endocrinol. Metab..

[78] McLaughlin T., Lamendola C., Liu A., Abbasi F. (2011). Preferential fat deposition in subcutaneous versus visceral depots is associated with insulin sensitivity. J. Clin. Endocrinol. Metab..

[79] Dumesic D.A., Winnett C., Lu G., Grogan T.R., Abbott D.H., Naik R., Chazenbalk G.D. (2023). Randomized Clinical Trial: Effect of Low-Dose Flutamide on Abdominal Adipogenic Function in Normal-Weight Polycystic Ovary Syndrome Women. Fertil. Steril..

[80] Dicker A., Ryden M., Naslund E., Muehlen I.E., Wiren M., Lafontan M., Arner P. (2004). Effect of testosterone on lipolysis in human pre-adipocytes from different fat depots. Diabetologia.

[81] Arner P. (2005). Effects of testosterone on fat cell lipolysis. Species differences and possible role in polycystic ovarian syndrome. Biochimie.

[82] Ek I., Arner P., Rydén M., Holm C., Thörne A., Hoffstedt J., Wahrenberg H. (2002). A unique defect in the regulation of visceral fat cell lipolysis in the polycystic ovary syndrome as an early link to insulin resistance. Diabetes.

[83] Zhou M.S., Wang A., Yu H. (2014). Link between insulin resistance and hypertension: What is the evidence from evolutionary biology?. Diabetol. Metab. Syndr..

[84] Samuel V.T., Petersen K.F., Shulman G.I. (2010). Lipid-induced insulin resistance: Unraveling the mechanism. Lancet.

[85] Chazenbalk G.D., Singh P., Irge D., Shah A., Abbott D.H., Dumesic D.A. (2013). Androgens inhibit adipogenesis during human adipose stem cell commitment to predipocyte formation. Steroids.

[86] Cristancho A.G., Lazar M.A. (2011). Forming functional fat: A growing understanding of adipocyte differentiation. Nat. Rev. Mol. Cell Biol..

[87] Tang Q.Q., Lane M.D. (2012). Adipogenesis: From stem cell to adipocyte. Annu. Rev. Biochem..

[88] Saponaro C., Gaggini M., Carli F., Gastaldelli A. (2015). The subtle balance between lipolysis and lipogenesis: A critical point in metabolic homeostasis. Nutrients.

[89] Romacho T., Elsen M., Rohrborn D., Eckel J. (2014). Adipose tissue and its role in organ crosstalk. Acta Physiol..

[90] Corbould A. (2007). Chronic testosterone treatment induces selective insulin resistance in subcutaneous adipocytes of women. J. Endocrinol..

[91] Rosenbaum D., Harber R.S., Dunaif A. (1993). Insulin resistance in polycystic ovary syndrome: Decreased expression of GLUT-4 glucose transporters in adipocytes. Am. J. Physiol..

[92] Faulds G., Rydén M., Ek I., Wahrenberg H., Arner P. (2003). Mechanisms behind lipolytic catecholamine resistance of subcutaneous fat cells in the polycystic ovarian syndrome. J. Clin. Endocrinol. Metab..

[93] Ek I., Arner P., Bergqvist A., Carlstrom KWahrenberg H. (1997). Impaired adipocyte lipolysis in nonobese women with the polycystic ovary syndrome: A possible link to insulin resistance?. J. Clin. Endocrinol. Metab..

[94] Mannerås-Holm L., Leonhardt H., Kullberg J., Jennische E., Odén A., Holm G., Hellström M., Lönn L., Olivecrona G., Stener-Victorin E. (2011). Adipose tissue has aberrant morphology and function in PCOS: Enlarged adipocytes and low serum adiponectin, but not circulating sex steroids, are strongly associated with insulin resistance. J. Clin. Endocrinol. Metab..

[95] Blouin K., Veilleux A., Luu-The V., Tchernof A. (2009). Androgen metabolism in adipose tissue: Recent advances. Mol. Cell. Endocrinol..

[96] Quinkler M., Sinha B., Tomlinson J.W., Bujalska I.J., Stewart P.M., Arlt W. (2004). Androgen generation in adipose tissue in women with simple obesity—A site-specific role for 17beta-hydroxysteroid dehydrogenase type 5. J. Endocrinol..

[97] O’Reilly M.W., Kempegowda P., Walsh M., Taylor A.E., Manolopoulos K.N., Allwood J.W., Semple R.K., Hebenstreit D., Dunn W.B., Tomlinson J.W. (2017). AKR1C3-Mediated Adipose Androgen Generation Drives Lipotoxicity in Women with Polycystic Ovary Syndrome. J. Clin. Endocrinol. Metab..

[98] Dumesic D.A., Tulberg A., McNamara M., Grogan T.R., Abbott D.H., Naik R., Lu G., Chazenbalk G.D. (2021). Serum Testosterone to Androstenedione Ratio Predicts Metabolic Health in Normal-Weight Polycystic Ovary Syndrome Women. J. Endocr. Soc..

[99] Fisch S.C., Nikou A.F., Wright E.A., Phan J.D., Leung K.L., Grogan T.R., Abbott D.H., Chazenbalk G.D., Dumesic D.A. (2018). Precocious Subcutaneous Abdominal Stem Cell Development to Adipocytes in Normal-Weight Polycystic Ovary Syndrome Women. Fertil. Steril..

[100] Leung K.L., Sanchita S., Pham C.T., Davis B.A., Okhovat M., Ding X., Dumesic P., Grogan T.R., Williams K.J., Morselli M. (2020). Dynamic changes in chromatin accessibility, altered adipogenic gene expression, and total versus de novo fatty acid synthesis in subcutaneous adipose stem cells of normal-weight polycystic ovary syndrome (PCOS) women during adipogenesis: Evidence of cellular programming. Clin. Epigenetics.

[101] Spalding K.L., Arner E., Westermark P.O., Bernard S., Buchholz B.A., Bergmann O., Blomqvist L., Hoffsted J., Naslund E., Britton T. (2008). Dynamics of fat cell turnover in humans. Nature.

[102] Tandon P., Wafer R., Minchin J.E. (2018). Adipose morphology and metabolic disease. J. Exp. Biol..

[103] Arner E., Westermark P.O., Spalding K.L., Britton T., Ryden M., Frisen J., Bernard S., Arner P. (2010). Adipocyte turnover: Relevance to human adipose tissue morphology. Diabetes.

[104] Longo M., Raciti G.A., Zatterale F., Parrillo L., Desiderio A., Spinelli R., Hammarstedt A., Hedjazifar S., Hoffmann J.M., Nigro C. (2018). Epigenetic modifications of the Zfp/ZNF423 gene control murine adipogenic commitment and are dysregulated in human hypertrophic obesity. Diabetologia.

[105] Nouws J., Fitch M., Mata M., Santoro N., Galuppo B., Kursawe R., Narayan D., Vash-Margita A., Pierpont B., Shulman G.I. (2019). Altered In Vivo Lipid Fluxes and Cell Dynamics in Subcutaneous Adipose Tissues Are Associated with the Unfavorable Pattern of Fat Distribution in Obese Adolescent Girls. Diabetes.

[106] Umano G.R., Shabanova V., Pierpont B., Mata M., Nouws J., Tricò D., Galderisi A., Santoro N., Caprio S. (2019). A low visceral fat proportion, independent of total body fat mass, protects obese adolescent girls against fatty liver and glucose dysregulation: A longitudinal study. Int. J. Obes..

[107] Brennan K.M., Kroener L.L., Chazenbalk G.D., Dumesic D.A. (2019). Polycystic Ovary Syndrome: Impact of Lipotoxicity on Metabolic and Reproductive Health. Obstet. Gynecol. Surv..

[108] Virtue S., Vidal-Puig A. (2008). It’s not how fat you are, it’s what you do with it that counts. PLoS Biol..

[109] Unger R.H., Clark G.O., Scherer P.E., Orci L. (2010). Lipid homeostasis, lipotoxicity and the metabolic syndrome. Biochim. Biophys. Acta.

[110] de Zegher F., Lopez-Bermejo A., Ibáñez L. (2009). Adipose tissue expandability and the early origins of PCOS. Trends Endocrinol. Metab..

[111] Shulman G.I. (2014). Ectopic fat in insulin resistance, dyslipidemia, and cardiometabolic disease. N. Eng. J. Med..

[112] Apridonidze T., Essah P.A., Iuorno M.J., Nestler J.E. (2005). Prevalence and characteristics of the metabolic syndrome in women with polycystic ovary syndrome. J. Clin. Endocrinol. Metab..

[113] Dokras A., Bochner M., Hollinrake E., Markham S., Vanvoorhis B., Jagasia D.H. (2005). Screening women with polycystic ovary syndrome for metabolic syndrome. Obstet. Gynecol..

[114] Ehrmann D.A., Liljenquist D.R., Kasza K., Azziz R., Legro R.S., Ghazzi M.N., PCOS/Troglitazone Study Group (2006). Prevalence and predictors of the metabolic syndrome in women with polycystic ovary syndrome. J. Clin. Endocrinol. Metab..

[115] Fazleen N.E., Whittaker M., Mamun A. (2018). Risk of metabolic syndrome in adolescents with polycystic ovarian syndrome: A systematic review and meta-analysis. Diabetes Metab. Syndr..

[116] Yang R., Yang S., Li R., Liu P., Qiao J., Zhang Y. (2016). Effects of hyperandrogenism on metabolic abnormalities in patients with polycystic ovary syndrome: A meta-analysis. Reprod. Biol. Endocrinol..

[117] Gambarin-Gelwan M., Kinkhabwala S.V., Schiano T.D., Bodian C., Yeh H.C., Futterweit W. (2007). Prevalence of nonalcoholic fatty liver disease in women with polycystic ovary syndrome. Clin. Gastroenterol. Hepatol..

[118] Macut D., Tziomalos K., Božić-Antić I., Bjekić-Macut J., Katsikis I., Papadakis E., Andrić Z., Panidis D. (2016). Non-alcoholic fatty liver disease is associated with insulin resistance and lipid accumulation product in women with polycystic ovary syndrome. Hum. Reprod..

[119] Vassilatou E., Vassiliadi D.A., Salambasis K., Lazaridou H., Koutsomitopoulos N., Kelekis N., Kassanos D., Hadjidakis D., Dimitriadis G. (2015). Increased prevalence of polycystic ovary syndrome in premenopausal women with nonalcoholic fatty liver disease. Eur. J. Endocrinol..

[120] Browning J.D., Horton J.D. (2004). Molecular mediators of hepatic steatosis and liver injury. J. Clin. Investig..

[121] Vassilatou E., Lafoyianni S., Vryonidou A., Ioannidis D., Kosma L., Katsoulis K., Papavassiliou E., Tzavara I. (2010). Increased androgen bioavailability is associated with non-alcoholic fatty liver disease in women with polycystic ovary syndrome. Hum. Reprod..

[122] Petta S., Ciresi A., Bianco J., Geraci V., Boemi R., Galvano L., Magliozzo F., Merlino G., Craxì A., Giordano C. (2017). Insulin resistance and hyperandrogenism drive steatosis and fibrosis risk in young females with PCOS. PLoS ONE.

[123] Jones H., Sprung V.S., Pugh C.J., Daousi C., Irwin A., Aziz N., Adams V.L., Thomas E.L., Bell J.D., Kemp G.J. (2012). Polycystic ovary syndrome with hyperandrogenism is characterized by an increased risk of hepatic steatosis compared to nonhyperandrogenic PCOS phenotypes and healthy controls, independent of obesity and insulin resistance. J. Clin. Endocrinol. Metab..

[124] Schwartz S.M., Kemnitz J.W., Howard C.F. (1993). Obesity in free-ranging rhesus macaques. Int. J. Obes. Relat. Metab. Disord..

[125] Raboin M.J., Letaw J., Mitchell A.D., Toffey D., McKelvey J., Roberts C.T., Curran J.E., Vinson A. (2019). Genetic Architecture of Human Obesity Traits in the Rhesus Macaque. Obesity.

[126] Kemnitz J.W., Goy R.W., Flitsch T.J., Lohmiller J.J., Robinson J.A. (1989). Obesity in male and female rhesus monkeys: Fat distribution, glucoregulation, and serum androgen levels. J. Clin. Endocrinol. Metab..

[127] Pound L.D., Kievit P., Grove K.L. (2014). The nonhuman primate as a model for type 2 diabetes. Curr. Opin. Endocrinol. Diabetes Obes..

[128] True C., Abbott D.H., Roberts C.T., Varlamov O. (2017). Sex Differences in Androgen Regulation of Metabolism in Nonhuman Primates. Adv. Exp. Med. Biol..

[129] Bodkin N.L., Alexander T.M., Ortmeyer H.K., Johnson E., Hansen B.C. (2003). Mortality and morbidity in laboratory-maintained Rhesus monkeys and effects of long-term dietary restriction. J. Gerontol. A Biol. Sci. Med. Sci..

[130] Bishop C.V., Takahashi D., Mishler E., Slayden O.D., Roberts C.T., Hennebold J., True C. (2021). Individual and combined effects of 5-year exposure to hyperandrogenemia and Western-style diet on metabolism and reproduction in female rhesus macaques. Hum. Reprod..

[131] Brown E., Ozawa K., Moccetti F., Vinson A., Hodovan J., Nguyen T.A., Bader L., López J.A., Kievit P., Shaw G.D. (2021). Arterial Platelet Adhesion in Atherosclerosis-Prone Arteries of Obese, Insulin-Resistant Nonhuman Primates. J. Am. Heart Assoc..

[132] Newman L.E., Testard C., DeCasien A.R., Chiou K.L., Watowich M.M., Janiak M.C., Pavez-Fox M.A., Sanchez Rosado M.R., Cooper E.B., Costa C.E. (2023). The biology of aging in a social world: Insights from free-ranging rhesus macaques. bioRxiv.

[133] Eisner J.R., Dumesic D.A., Kemnitz J.W., Colman R.J., Abbott D.H. (2003). Increased adiposity in female rhesus monkeys exposed to androgen excess during early gestation. Obes. Res..

[134] Barnett D.K., Abbott D.H. (2003). Reproductive adaptations to a large-brained fetus open a vulnerability to anovulation similar to polycystic ovary syndrome. Am. J. Hum. Biol..

[135] Bruns C.M., Baum S.T., Colman R.J., Dumesic D.A., Eisner J.R., Jensen M.D., Whigham L.D., Abbott D.H. (2007). Prenatal androgen excess negatively impacts body fat distribution in a nonhuman primate model of polycystic ovary syndrome. Int. J. Obes..

[136] Zhou R., Bruns C.M., Bird I.M., Kemnitz J.W., Goodfriend T.L., Dumesic D.A., Abbott D.H. (2007). Pioglitazone improves insulin action and normalizes menstrual cycles in a majority of prenatally androgenized female rhesus monkeys. Reprod. Toxicol..

[137] Abbott D.H., Barnett D.K., Bruns C.M., Dumesic D.A. (2005). Androgen excess fetal programming of female reproduction: A developmental aetiology for polycystic ovary syndrome?. Hum. Reprod. Update.

[138] Keller E., Chazenbalk G.D., Aguilera P., Madrigal V., Grogan T., Elashoff D., Dumesic D.A., Abbott D.H. (2014). Impaired preadipocyte differentiation into adipocytes in subcutaneous abdominal adipose of PCOS-like female rhesus monkeys. Endocrinology.

[139] Barrett E.S., Hoeger K.M., Sathyanarayana S., Abbott D.H., Redmon J.B., Nguyen R.H.N., Swan S.H. (2018). Anogenital distance in newborn daughters of women with polycystic ovary syndrome indicates fetal testosterone exposure. J. Dev. Orig. Health Dis..

[140] Sánchez-Ferrer M.L., Mendiola J., Hernández-Peñalver A.I., Corbalán-Biyang S., Carmona-Barnosi A., Prieto-Sánchez M.T., Nieto A., Torres-Cantero A.M. (2017). Presence of polycystic ovary syndrome is associated with longer anogenital distance in adult Mediterranean women. Hum. Reprod..

[141] Abbott A.D., Colman R.J., Tiefenthaler R., Dumesic D.A., Abbott D.H. (2012). Early-to-mid gestation fetal testosterone increases right hand 2D:4D finger length ratio in polycystic ovary syndrome-like monkeys. PLoS ONE.

[142] Tata B., Mimouni N.E.H., Barbotin A.L., Malone S.A., Loyens A., Pigny P., Dewailly D., Catteau-Jonard S., Sundström-Poromaa I., Piltonen T.T. (2018). Elevated prenatal anti-Müllerian hormone reprograms the fetus and induces polycystic ovary syndrome in adulthood. Nat. Med..

[143] Mimouni N.E.H., Paiva I., Barbotin A.L., Timzoura F.E., Plassard D., Le Gras S., Ternier G., Pigny P., Catteau-Jonard S., Simon V. (2021). Polycystic ovary syndrome is transmitted via a transgenerational epigenetic process. Cell Metab..

[144] Abbott D.H., Tarantal A.F., Dumesic D.A. (2009). Fetal, infant, adolescent and adult phenotypes of polycystic ovary syndrome in prenatally androgenized female rhesus monkeys. Am. J. Primatol..

[145] Susa J.B., Neave C., Sehgal P., Singer D.B., Zeller W.P., Schwartz R. (1984). Chronic hyperinsulinemia in the fetal rhesus monkey. Effects of physiologic hyperinsulinemia on fetal growth and composition. Diabetes.

[146] Filippou P., Homburg R. (2017). Is foetal hyperexposure to androgens a cause of PCOS?. Hum. Reprod. Update.

[147] Palomba S., Russo T., Falbo A., Di Cello A., Tolino A., Tucci L., La Sala G.B., Zullo F. (2013). Macroscopic and microscopic findings of the placenta in women with polycystic ovary syndrome. Hum. Reprod..

[148] Zhang Q., Bao Z.K., Deng M.X., Xu Q., Ding D.D., Pan M.M., Xi X., Wang F.F., Zou Y., Qu F. (2020). Fetal growth, fetal development, and placental features in women with polycystic ovary syndrome: Analysis based on fetal and placental magnetic resonance imaging. J. Zhejiang Univ. Sci. B.

[149] Hochberg A., Mills G., Volodarsky-Perel A., Nu T.N.T., Machado-Gedeon A., Cui Y., Shaul J., Dahan M.H. (2023). The impact of polycystic ovary syndrome on placental histopathology patterns in in-vitro fertilization singleton live births. Placenta.

[150] Kuo K., Roberts V.H.J., Gaffney J., Takahashi D.L., Morgan T., Lo J.O., Stouffer R.L., Frias A.E. (2019). Maternal High-Fat Diet Consumption and Chronic Hyperandrogenemia Are Associated with Placental Dysfunction in Female Rhesus Macaques. Endocrinology.

[151] Abbott D.H., Bruns C.R., Barnett D.K., Dunaif A., Goodfriend T.L., Dumesic D.A., Tarantal A.F. (2010). Experimentally induced gestational androgen excess disrupts glucoregulation in rhesus monkey dams and their female offspring. Am. J. Physiol. Endocrinol. Metab..

[152] Harnois-Leblanc S., Hernandez M.I., Codner E., Cassorla F., Oberfield S.E., Leibel N.I., Mathew R.P., Ten S., Magoffin D.A., Lane C.J. (2022). Profile of Daughters and Sisters of Women with Polycystic Ovary Syndrome: The Role of Proband’s Glucose Tolerance. J. Clin. Endocrinol. Metab..

[153] Hanem L.G.E., Salvesen Ø., Madsen A., Sagen J.V., Mellgren G., Juliusson P.B., Carlsen S.M., Vanky E., Ødegård R. (2021). Maternal PCOS status and metformin in pregnancy: Steroid hormones in 5–10 years old children from the PregMet randomized controlled study. PLoS ONE.

[154] Maliqueo M., Galgani J.E., Pérez-Bravo F., Echiburú B., de Guevara A.L., Crisosto N., Sir-Petermann T. (2012). Relationship of serum adipocyte-derived proteins with insulin sensitivity and reproductive features in pre-pubertal and pubertal daughters of polycystic ovary syndrome women. Eur. J. Obstet. Gynecol. Reprod. Biol..

[155] Sir-Petermann T., Maliqueo M., Codner E., Echiburú B., Crisosto N., Pérez V., Pérez-Bravo F., Cassorla F. (2007). Early metabolic derangements in daughters of women with polycystic ovary syndrome. J. Clin. Endocrinol. Metab..

[156] Warren W.C., Harris R.A., Haukness M., Fiddes I.T., Murali S.C., Fernandes J., Dishuck P.C., Storer J.M., Raveendran M., Hillier L.W. (2020). Sequence diversity analyses of an improved rhesus macaque genome enhance its biomedical utility. Science.

